# INTERGENERATIONAL WORKSHOP TO EMPOWER OLDER ADULTS IN SMART TECHNOLOGIES

**DOI:** 10.1093/geroni/igae098.1641

**Published:** 2024-12-31

**Authors:** Duo Wei, Joseph Morales, David Burdick

**Affiliations:** Stockton University, Galloway, New Jersey, United States; Stockton University, Galloway, New Jersey, United States; Stockton University, Galloway, New Jersey, United States

## Abstract

Intergenerational programming, as outlined by the National Council on the Aging, involves those “activities or programs that increase cooperation, interaction, or exchange between any two generations. It involves the sharing of skills, knowledge or experience between old and young.” The overall goal of the intergenerational service learning project of the Medical Informatics class is to promote learning between university students and older adults in the community, and to put the concept of lifelong learning into practice. In the Medical Informatics class, students collaborate in multiple groups to organize technology training workshops. These workshops are tailored to educate older adult community members on a range of technology-related subjects, including utilizing smartphones and AI technologies for social engagement, entertainment, and health management, with the aim of enhancing health outcomes. The training sessions have been held at various venues across the South Jersey region, including AtlantiCare, Center for Family Services, Hammonton Canoe Club, Capital Health Medical Center, Four Seasons Clubhouse at Smithville, and others. The effectiveness of the intergenerational programming has been assessed through student feedback, surveys of the older participants, student faculty/course evaluations, and media coverage. Results indicate that this service learning initiative is a valuable approach in fostering a culture of learning among university students and community partners alike. University students act as mentors to older adults, while also benefiting from the diverse perspectives shared by the older generation, thus enriching their learning experience. Moreover, this collaborative effort nurtures stronger bonds between communities and the university, promoting mutual understanding and support.

